# Automatic Reconstruction of Mitochondria and Endoplasmic Reticulum in Electron Microscopy Volumes by Deep Learning

**DOI:** 10.3389/fnins.2020.00599

**Published:** 2020-07-21

**Authors:** Jing Liu, Linlin Li, Yang Yang, Bei Hong, Xi Chen, Qiwei Xie, Hua Han

**Affiliations:** ^1^National Laboratory of Pattern Recognition, Institute of Automation, Chinese Academy of Sciences, Beijing, China; ^2^School of Artificial Intelligence, School of Future Technology, University of Chinese Academy of Sciences, Beijing, China; ^3^School of Life Science and Technology, ShanghaiTech University, Shanghai, China; ^4^Data Mining Lab, Beijing University of Technology, Beijing, China; ^5^CAS Center for Excellence in Brain Science and Intelligence Technology, Shanghai, China

**Keywords:** mitochondria, endoplasmic reticulum, electron microscopes, segmentation, 3D reconstruction

## Abstract

Together, mitochondria and the endoplasmic reticulum (ER) occupy more than 20% of a cell's volume, and morphological abnormality may lead to cellular function disorders. With the rapid development of large-scale electron microscopy (EM), manual contouring and three-dimensional (3D) reconstruction of these organelles has previously been accomplished in biological studies. However, manual segmentation of mitochondria and ER from EM images is time consuming and thus unable to meet the demands of large data analysis. Here, we propose an automated pipeline for mitochondrial and ER reconstruction, including the mitochondrial and ER contact sites (MAMs). We propose a novel recurrent neural network to detect and segment mitochondria and a fully residual convolutional network to reconstruct the ER. Based on the sparse distribution of synapses, we use mitochondrial context information to rectify the local misleading results and obtain 3D mitochondrial reconstructions. The experimental results demonstrate that the proposed method achieves state-of-the-art performance.

## 1. Introduction

In eukaryotic cells, mitochondria and the endoplasmic reticulum (ER) together occupy more than 20% of the cell volume. Evidence suggests that mitochondrial and ER morphology changes can have severe consequences for cell physiological or pathological functions, such as apoptosis, Ca^2+^ homeostasis, and metabolite processing (Karbowski and Youle, 2003; Twig et al., 2008; Bhatti et al., 2017). Mitochondria–ER plasma membrane (MAM) contacts are particularly abundant in cell bodies and are also important for multiple physiological functions (Marchi et al., 2014). Recently, emerging evidence has documented that mitochondria, ER, and MAM abnormalities or dysfunctions can result in various neurodegenerative disorders, such as Alzheimer's disease, Parkinson's disease, and amyotrophic lateral sclerosis (Manfredi and Kawamata, 2016; Liu and Zhu, 2017; Rodriguez-Arribas et al., 2017).

The three-dimensional (3D) ultrastructure of mitochondria and ER at high resolutions requires large 3D reconstructions to be generated via electron microscopy (EM) (Vincent et al., 2016; Hirabayashi et al., 2017; Krols et al., 2018; Delgado et al., 2019). With the rapid development of large-scale electron microscopy (EM) technology, the brain imaging methods have undergone tremendous changes. Manual contouring and reconstruction of the 3D ultrastructures of these organelles from high-resolution EM images has previously been accomplished in biological studies, such as glycogen granules (Agus et al., 2019) and mitochondria (Calì et al., 2019). However, manual annotation alone is insufficient to match the speed of data acquisition and meet the data analysis demands. Hence, developing an automatic algorithm to detect mitochondria and ER from EM images is both necessary and urgent.

Unlike ER segmentation, which has been less studied thus far, the detection and segmentation of mitochondria has been a hot topic in the neuroscience and computer vision fields. Nevertheless, the complex textures as well as similar ultrastructures in EM images have made the segmentation of mitochondria a challenging problem. Recently, variety of methods have been developed to automatically detect and segment mitochondria (Liu et al., 2018; Xiao et al., 2018; Xie et al., 2018b). GentleBoost classifier was trained for detecting mitochondria based on textural features (Vitaladevuni et al., 2008). Narasimha et al. (2009) utilized multiple classifiers to localize and segment mitochondria jointly in 3D images. Lucchi et al. (2012b) presented an automated approach with consideration of 3D shape cues for segmentation of mitochondria, and this method greatly reduced computational complexity by operating on super voxels instead of voxels. And then they improved classification accuracy by using context-based features and modeling the double membrane that encloses mitochondria (Lucchi et al., 2014). Jorstad and Fua (2015) iteratively refined the boundaries of mitochondria surfaces, starting from rough prediction provided by a machine learning-based method. In addition, there are some methods introducing graphical models into segmentation of mitochondria and achieving promising results, such as Markov Random Fields (MRFs) and Conditional Random Fields (CRFs) (Lucchi et al., 2012a, 2013; Márquez-Neila et al., 2014).

However, all the aforementioned works require handcrafted mitochondrial features as the kernel of the algorithm. The wide success of the convolutional neural network (CNN) when applied to image processing tasks has proven that these models can learn powerful feature representations (Huang et al., 2018; Xie et al., 2018a). In the field of computer vision, instance segmentation, which involves the automatic detection of all objects appearing in an image and precise delineation of each instance, has undergone significant improvements based on deep learning models in recent years. We briefly review some of the most significant works below. Mask R-CNN (He et al., 2017) segments objects based on bounding boxes produced by CNNs. Concretely, Mask R-CNN extends Faster R-CNN by adding a parallel branch to predict a segmentation mask for each region of interest (RoI). In addition to the proposal-based methods, recurrent networks have gradually been introduced to solve the instance segmentation problem. Reversible Recursive Instance-level Object Segmentation (R2-IOS) (Liang et al., 2016) is composed primarily of two subnetworks. A reversible proposal refinement subnetwork is used to refine the object bounding boxes, while an instance-level segmentation subnetwork predicts the object mask for each proposal. The flooding filling network (FFN, Januszewski et al., 2018) utilizes a recurrent convolutional network to delineate a single object; it takes an object image channel and an object mask channel as inputs. The output mask channel of the FFN serves as the input mask channel for the next iteration, providing an explicit snapshot of the current segmentation state. One major challenge in FFNs is determining the initial state of the object mask, which is called a seed.

**Figure 1:**
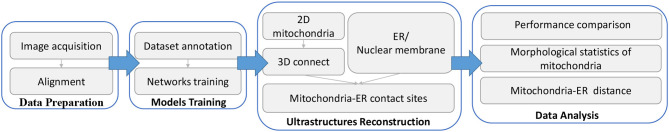
Workflow of our proposed method. Briefly, there are four main steps in the complete method. (1) Data preparation: First, serial images are obtained by SEM and then aligned to create 3D image stacks using a non-linear registration method. (2) Model training: From the aligned data, we annotate some images to form a training dataset, and then separately train two networks to process mitochondria and ER. (3) Ultrastructure reconstruction: 2D mitochondria were predicted using the trained network and connected to form 3D mitochondria. The ER is segmented by the trained network. Then, we detect mitochondria-ER contact sites from the above results. (4) Data analysis: We measure the performance of our method and calculate biological measurements to conduct various analyses.

In this paper, we aim to develop an automated pipeline for detecting mitochondria and ER to seek morphological characteristics in different domains. The workflow of the entire pipeline is illustrated in Figure 1. The main contributions of this work are as follows:

1. We present an efficient, fully CNN to reconstruct the ER from EM images. To our knowledge, this is the first work that applies a deep network to ER segmentation.
2. We propose a novel recurrent network that iteratively refines mitochondrial segmentation. The detection subnetwork generates bounding boxes that are passed to the segmentation subnetwork as the initial seeds. Then, the segmentation subnetwork recursively identifies the mitochondrial boundaries.
3. For the highly anisotropic EM images, we introduce an efficient 2D-to-3D approach to reconstruct mitochondria that first obtains 2D segmentation results utilizing the recurrent network; then, morphological processing and mitochondrial context information are used to rectify local misleading results. Finally, a 3D connection algorithm is applied to obtain the 3D mitochondrial structures.
4. We reconstructed all the mitochondria and the ER on two EM image stacks with volumes up to ~ 28,628 μm^3^ and found 4 different structural features of mitochondria-ER contact sites.

## 2. Materials and Methods

### 2.1. Materials

The mouse cortex sample used in this study was acquired by the Institute of Neuroscience, Chinese Academy of Sciences. The tissue block was automatically cut into serial sections with thicknesses of approximately 50 nm. Then, the sections were imaged by the Institute of Automation at the Chinese Academy of Sciences using a scanning electron microscope (SEM) (Zeiss Supra55) with a resolution of 3 nm × 3 nm and a dwell time of 1.5 μs. The SEM images were acquired using secondary electron detection (9 kV accelerating potential, and at a working distance of ~ 6.0 mm). A total of 511 image planes with a size of 15,000 × 8,300 were acquired, yielding 59.25 GB of data.

### 2.2. Image Preprocessing

**Figure 2:**
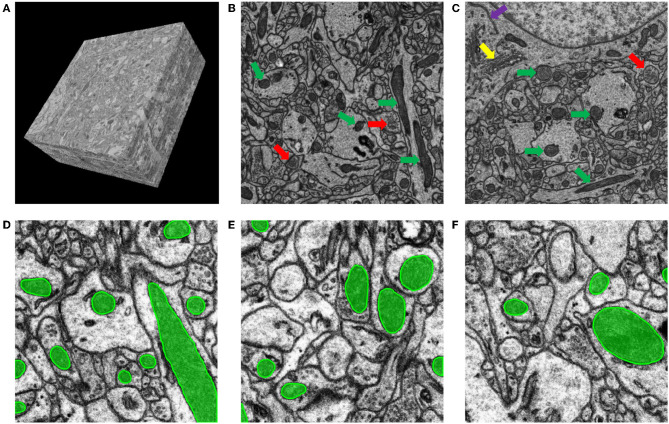
Mouse cortex neural tissue acquired by ATUM-SEM. **(A)** An example of an aligned image stack that covers approximately 20 × 20 × 10 μm through the ATUM-SEM method. **(B,C)** Examples of mitochondria and other ultrastructures. The green arrows indicate mitochondria; the red arrows indicate vesicles; the yellow arrow indicates the Golgi body, and the purple arrow indicates the endoplasmic reticulum. **(D–F)** Examples of mitochondria segmentation in our training dataset.

As mentioned above, the imaging data were obtained by SEM. The SEM technique is non-destructive, which means that the specimens can be imaged more than once. However, because of the section collection method, arbitrary angle rotations and some distortions are inevitable. Hence, image alignment is important to correct these errors and obtain a sequential image stack. We adopted the method in (Chen et al., 2018) for image registration. First, we used the scale invariant feature transform (SIFT, Liu et al., 2008) to detect corresponding landmarks across adjacent sections. Then, the wrinkled areas were annotated manually. Finally, we used a modified moving-least-squares (MLS, Schaefer et al., 2006) deformation algorithm to register adjacent sections with wrinkles. This algorithm reflects the discontinuity around wrinkle areas while maintaining smoothness in other regions. A registered image stack is shown in Figure 2.

In addition, some differences exist in the distribution of grayscale values in the EM images. To reduce the network training difficulty, we preprocessed the raw images using a histogram matching method to maintain grayscale consistency.

### 2.3. Proposed Network for Mitochondria

#### 2.3.1. Network Architecture

Mask R-CNN (He et al., 2017) was proposed to solve the problem of instance segmentation based on segment proposals. It produces candidate object regions through a region proposal network (RPN). Based on the proposals from the RPN, R-CNN conducts further classification and regression. In contrast to Faster R-CNN (Ren et al., 2015), Mask R-CNN predicts object masks in end-to-end fashion by adding a mask branch. In combination with the feature pyramid network (FPN, Lin et al., 2017) in the backbone network, Mask R-CNN can extract features from different levels of the feature pyramid based on the scale of each RoI. This mechanism is able to exploit more detailed features to obtain finer segmentation results for small objects.

**Figure 3:**
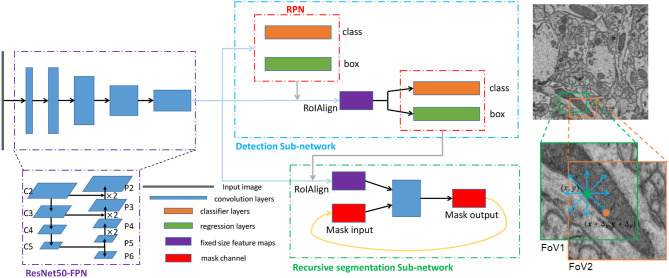
The architecture and training scheme of the proposed network for segmenting mitochondria. **Left**: The three dotted boxes represent the backbone network (purple), the detection subnetwork (blue) and the recursive segmentation subnetwork (green). The black block, blue blocks, orange blocks, green blocks, purple blocks, and red blocks indicate the input image, convolution layers, classification layers, regression layers, and the fixed-size feature maps obtained from the RoIAlign and mask channels, respectively. **Right**: A simplified execution scheme for segmenting mitochondria. The green box indicates the outputs from the detection subnetwork, which may be smaller than the ground truth. After the first iteration of the segmentation subnetwork, the eight prediction directions are checked, and the direction is the input for the next iteration. In this example, the position in the orange circle is selected, and then the field of view (FoV) moves to the orange box.

However, mitochondria vary widely in size and shape. In addition to the common elliptical mitochondria, some are long and narrow, and some are curly. A Mask R-CNN model with an FPN backbone is unsuitable for this situation. Specifically, the bounding boxes that R-CNN predicts are always smaller than the true bounding boxes for larger mitochondria, which leads to incomplete segmentation due to the series structure of the mask branch and R-CNN. Inspired by the scheme proposed in FFN (Januszewski et al., 2018), we propose to refine the segmentation results by moving the field of view (FoV) of the mask branch to extend the detection boxes, which is implemented by introducing an input mask that preserves the previous segmentation state. In the following subsections, we describe the key components of the proposed network (see Figure 3) in detail, including the detection subnetwork and the recursive segmentation subnetwork.

**Figure 4:**
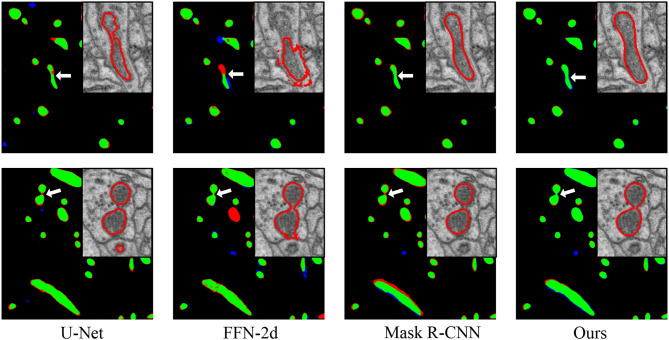
Performance comparisons with the baseline approaches. From left to right: comparisons to the manual ground truth from U-Net, FFN-2d, Mask R-CNN and our proposed network. In each comparison image, green pixels represent true positives (TP), blue pixels represent false positives (FP), red pixels represent false negatives (FN), and black pixels represent true negatives (TN). The insert at the top right shows enlarged details pointed to by the white arrow. Qualitatively, the segmentation by the proposed network contains fewer false positives than that of FFN-2d and more true positives than those of U-Net and Mask R-CNN.

##### 2.3.1.1. Detection subnetwork

Due to the relatively simple characteristics of mitochondria, a very deep network may cause overfitting; moreover, a simpler network will have considerably lower computational costs. Therefore, we adopt ResNet50 (He et al., 2016) as the backbone network. Considering the substantial variance in mitochondrial size, we utilize a feature pyramid network (Lin et al., 2017) to explore features at different scales. Concretely, an FPN uses a top-down architecture with lateral connections to build an in-network feature pyramid from a single-scale input. The main task of the RPN is to produce the candidate object regions. At every feature level of FPN, we use 3 ratios, yielding 3 anchors at each location of the convolutional layer. The RPN predicts a score and a bounding-box regression for each anchor. Then, RoI features are extracted from different levels of the feature pyramid based on their scales. To avoid misalignments between the RoI and the extracted features, we use RoIAlign to avoid any quantization operations. Finally, R-CNN performs further classification and regression based on the RoI features from RPN. The outputs of the R-CNN are then used as the initial inputs of the recursive segmentation subnetwork.

##### 2.3.1.2. Recursive segmentation subnetwork

The main role of the recursive segmentation subnetwork is to achieve a precise depiction of the mitochondria detected by the detection subnetwork. The recursive segmentation subnetwork takes an RoI feature and an object mask as inputs and predicts the probability map of the object with the focus. The input object mask is initialized by assigning the center position of the detection box an active value and assigning the other positions zero. This serves to show that the center pixel may be part of the target. Then, the subnetwork runs a forward pass to obtain a segmentation mask for the current FoV. Due to the existence of incomplete detection boxes, we introduce a mechanism for moving the FoV to extend the object via the subnetwork iterations. Note that the input object mask in the next iteration uses the updated probability map based on the output of the preceding iteration. This approach provides information about the current focus target.

The specific architecture of the segmentation refinement subnetwork is shown in Figure 3. Because the detection boxes have different sizes, we still use RoIAlign to obtain fixed-size feature maps from the corresponding level of the feature pyramid; these function as the feature channel of the inputs. In this study, we set the fixed size to 33 × 33. To combine the two inputs, we simply concatenate them. The subsequent operation includes four convolution layers with a ReLU: each convolution has a kernel size of 3 × 3 and uses the same mode to guarantee an identical output size. Before each ReLU, we utilize batch normalization (BN) to accelerate convergence during the training process. In the final layer, we use a 1 × 1 convolution to reduce the number of channels to the desired number of classes, which is one in our case. We do not have many convolution operations in this subnetwork, and one of the reasons is that the input features are extracted from the shared feature pyramid, which already possesses high-level abstract information. Reusing these shared features greatly simplifies the training process.

#### 2.3.2. Training

The two subnetworks are trained separately to learn mitochondrial feature representations to obtain the detection and segmentation results.

For the detection subnetwork, the implementation details are similar to those of Lin et al. (2017). We obtain the last residual block of each stage from ResNet50 as one pyramid level of the bottom-up pathway in FPN, denoted as C_2_, C_3_, C_4_, and C_5_. We upsample the feature maps and leverage lateral connections to form the top-down pathways P_2_, P_3_, P_4_, and P_5_, as shown in Figure 3. Notably, we also introduce pathway P_6_ to enlarge the range of the scale of anchors by simply subsampling P_5_. P_6_ is used only in the RPN to predict candidate regions. From the high-level to the low-level feature maps, the corresponding anchor scales are {32^2^, 64^2^, 128^2^, 256^2^, 512^2^}, and each scale has three ratios, {1 : 1, 1 : 2, 2 : 1}. The RPN generates RoIs of various sizes; we loop through all the candidate RoIs and extract features from the pyramid levels according to their scales. This strategy helps ensure that larger objects obtain stronger semantic features and that smaller objects obtain more detailed features.

This paper defines a multitask loss as

(1) $$\begin{array}{r} L=L_{cls}^{rpn}+L_{box}^{rpn}+L_{cls}^{r-cnn}+L_{box}^{r-cnn}, \end{array}$$

Based on the aforementioned settings, we trained the detection subnetwork with a backpropagation algorithm and saved the weight parameters of the network. Then, we initialized the recursive segmentation subnetwork with the trained model to share the FPN backbone features.

To optimize the segmentation subnetwork, we use cross-entropy loss, which can be represented as follows:

(2) $$\begin{array}{r} L_{mask}=\underset{i}{\sum }-\left[y_{i}*log\left(p_{i}\right)+\left(1-y_{i}\right)*log\left(1-p_{i}\right)\right], \end{array}$$

where *y* denotes the ground-truth label, and *p* denotes the prediction for each pixel, which is the activation value of the sigmoid. *i* is the pixel index.

During the second training stage, the training samples are generated randomly. Specifically, we first generate RoI proposals to simulate the detection results of the detection subnetwork. Then, the RoI features are extracted from the corresponding level of pyramid features according to the scale of the bounding box, and the RoI mask is extracted from a mask we maintain during training to update the probability map of the iterative procedure. To fit the size of the input channel, we resize the RoI mask using the nearest bilinear interpolation algorithm. Similarly, the ground-truth mask should be resized to the same size. Here, we use nearest neighbor interpolation to guarantee a binary value in the ground truth.

The movement of the FoV is restricted to one step away from the current position to simplify the training process. After one forward pass of a training sample, the eight directions relative to the current position are checked to seek the new positions that the network will move to next (see Figure 3). We set a movement step (Δ_*x*_, Δ_*y*_) = (8, 8) in the fixed-size (33, 33) object mask to inform the movement. That is, first, the center position of the current FoV is denoted as (*x, y*); then, we sequentially check the eight new positions (*x* + Δ_*x*_, *y*), (*x* + Δ_*x*_, *y* + Δ_*y*_), (*x, y* + Δ_*y*_), (*x* − Δ_*x*_, *y* + Δ_*y*_), (*x* − Δ_*x*_, *y*), (*x* − Δ_*x*_, *y* − Δ_*y*_), (*x, y* − Δ_*y*_), (*x* + Δ_*x*_, *y* − Δ_*y*_). During the inspection, if the predicted probability is greater than or equal to the moving threshold (*T*_*move*_ = 0.9), that point becomes the new center of the FoV in the next iteration. This process is repeated until all possible positions have been traversed.

#### 2.3.3. Inference

The inference process is also divided into two steps. First, the proposals (*x*_1_, *y*_1_, *x*_2_, *y*_2_) are obtained by running the detection subnetwork. Then, the recursive segmentation subnetwork takes these boxes as inputs and refines the segmentation results recursively.

Compared with training, the inference scheme of the segmentation subnetwork has the following characteristics. To extend to the complete mitochondria, the inference is not limited to only one step in the eight directions; instead, a queue is maintained to preserve the new positions. Before a new iteration, a position is popped; then, new positions are computed after a forward pass and pushed into the queue in descending order.

If we denote the width and height of the current box as (*w, h*) and the size of the input object mask as (*s, s*), then the movement vector is computed as follows:

(3) $$\begin{array}{r} \left({\Delta_{x}}^{\prime },{\Delta_{y}}^{\prime }\right)=\left(\frac{\Delta_{x}\;\cdot \;w}{s},\frac{\Delta_{y}\;\cdot \;h}{s}\right). \end{array}$$

This simple strategy results in the process taking larger steps for larger objects and smaller steps for smaller objects, which helps to solve the inefficiency problem to a certain extent.

### 2.4. Proposed Network for ER

**Figure 5:**
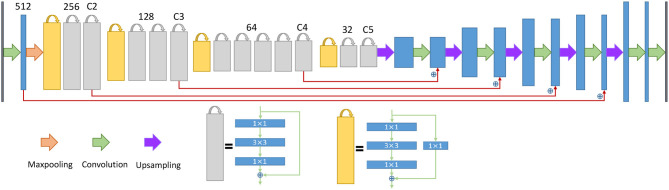
The architecture of the neural network for the endoplasmic reticulum. The yellow blocks and gray blocks denote two different residual blocks, and the numbers above the blocks indicate the number of feature map channels. The blue plus signs indicate a sum operation rather than concatenation.

To explore the contact sites between the ER and mitochondria, we need to obtain the morphologies of the ER. We also observed that the nuclear membrane was incorrectly detected if we attempted to segment the ER only. Therefore, we decided to segment the ER and the nuclear membrane as an auxiliary task using one deep neural network, which promotes the ER segmentation performance. Additionally, the inner nuclear membrane is labeled in the training samples as a third category used to distinguish the ER and nuclear membrane in postprocessing. Then, we build a fully convolutional network based on ResNet50 (He et al., 2016) to classify all the pixels in one image. The network contains an analysis and a synthesis path similar to the architecture of U-Net (see Figure 5). The first six resolution steps in the analysis path are taken from the first six stages of ResNet50. In the synthesis path, each resolution step is upsampled first, followed by a 3 × 3 convolution layer and batch normalization (BN). Then, the layer is merged with the equal resolution from the analysis path by a pixelwise addition operation, followed by a rectified linear unit (ReLU). Using this strategy, the localization and contextual information can be finely balanced. In the final layer, a 1 × 1 convolution layer is used to map the feature channel to the desired number of classes, which is 3 in our case.

**Figure 6:**
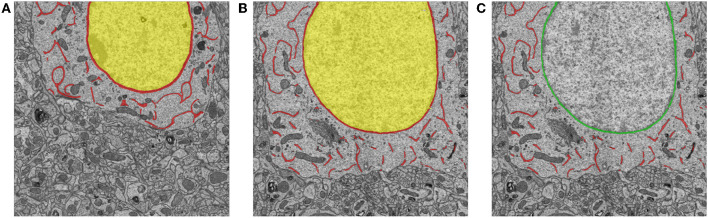
Examples of training samples and predictions for ER segmentation. **(A)** An example of manual annotations of the nucleus (yellow), ER, and the nuclear membrane (red). **(B,C)** An inference from the trained neural network and the corresponding relabeled result. The red and green pixels indicate the ER and the nuclear membrane, respectively.

During inference, each pixel is classified with the class that has the maximum value in the three-dimensional classification vector. To differentiate ER from the nuclear membrane, we mark the connected components that are adjacent to the interior of the nuclear membrane as the nuclear membrane; then, the remaining components are labeled as the ER. To illustrate this process of separating ER from the nuclear membrane, Figure 6 shows an example that includes a prediction from the network and the corresponding relabeled result in.

### 2.5. Training and Testing Datasets

As noted previously, we acquired an SEM dataset with a voxel resolution size of 3 nm × 3 nm × 50 nm.

To create a dataset for ER segmentation, we manually labeled 60 serial sections with sizes of 4,096 × 6,344, used 49 slices as the training data, and used the remainder as testing data. The pixels were labeled with three categories: ER, nuclear membrane, and interior of nuclear membrane or background. Figure 6 shows a manually annotated sample.

To create a dataset for segmentation of mitochondria, we extracted 15 slices as a training dataset, each of which has a size of 7,168 × 7,168; then, 5 slices of the same size were used as a test dataset to evaluate the algorithm performance. The ground truth of the mitochondria dataset was annotated by experienced students using Trakem2 software (Cardona et al., 2012). Figure 2 shows some specific examples from our annotated ATUM-SEM dataset. In addition, to ensure a fair comparison with existing methods, we conducted experiments on the FIB-SEM dataset, which has been widely used for mitochondrial segmentation. The FIB-SEM dataset contains a training volume and a testing volume taken from the CA1 hippocampus region of the brain. Each volume consists of 165 slices with a resolution of 5 × 5 × 5 nm.

Deep learning requires a considerable amount of training data to avoid overfitting problems. Therefore, we enlarged the training dataset using data augmentation techniques, including random rotation, random flipping and adding random noise. The data augmentation process was conducted online.

### 2.6. Experimental Setup

All the proposed networks were implemented using the Keras open-source deep learning library (Chollet et al., 2015), with the TensorFlow library as the backend. Due to GPU memory constraints, the original images were cut into smaller images as inputs. We trained all the networks using stochastic gradient descent. However, the related training parameters were slightly different between the networks for mitochondria and ER. For the mitochondrial network, the momentum was set to 0.9, the weight decay was set to 0.0001, and the learning rate was initially set to 0.001 and then decreased by a factor of 10 whenever the learning process stagnated. The ER network was trained using a learning rate of 0.01 and a momentum of 0.9. All the training and testing tasks were conducted on a server equipped with an Intel i7 CPU, 512 GB of main memory, and a Tesla K40 GPU.

### 2.7. Method for Reconstructing Mitochondria

Because we employed a refinement subnetwork to obtain more precise segmentation results, many trivial false segmentations will appear in the 2D slices. Therefore, a suitable postprocessing method is necessary to improve accuracy. This section focuses on how the segmentation results are optimized to further improve the performance while obtain 3D mitochondria. Note that the mitochondrial sizes are far larger than the resolution in the z-direction. We utilize the multilayer information fusion algorithm proposed by Li et al. (2018) to reconstruct the mitochondria in 3D and discard mitochondria whose “length” (the number of occurrences in the z-direction) is less than *L* (e.g., 15).

For completeness, we note that there is no need to conduct 3D connections for the ER and nuclear membrane. We labeled three cell bodies manually and this analysis is restricted to the cell body only.

### 2.8. Measurement of Biological Statistics

To obtain the cross sections, the first task is to obtain the skeleton of the mitochondrion. We first obtain the main skeleton, excluding the tiny branches, using ImageJ software. However, the anisotropy of the data causes the main skeleton to be a rough line. We smooth this skeleton by applying polynomial fitting. Finally, we take some points on this skeleton line and determine the perpendicular planes. Thus, the cross sections of one mitochondrion are the intersections of the perpendicular planes and the 3D mitochondrion. Using this approach, we measured the area and perimeter of the cross section.

To obtain the minimum distances between mitochondria and the ER, we simply compute all the point pairs and find the smallest values. To reduce the computational complexity, we downsampled the original images by a factor of 4 in the X and Y directions and a factor of 2 in the Z direction. Additionally, we reduced the number of points by edge extraction.

## 3. Results

### 3.1. Performance Comparisons

In this subsection, we focus primarily on the segmentation performance and the computational costs of the proposed method. For completeness, we do not measure the segmentation performance of ER because no method for doing exists at present.

#### 3.1.1. Evaluation Metrics

To quantify the pixelwise segmentation quality, we select the following metrics: *Jaccard index, Accuracy, Precision, Recall, F-score*, and *Dice coefficient*; these are the common criteria used in image segmentation tasks. All these measures are calculated based on combinations of the number of true positives (TPs), true negatives (TNs), false positives (FPs), and false negatives (FNs) of the predicted pixels. The values of all the criteria range from 0 to 1, where 0 indicates no coincidence and 1 denotes total coincidence.

*Jaccard index* is defined as the ratio of the area of the intersection and the area of the union between the ground truth and the segmentation result. *Dice coefficient* computes the spatial overlap between the ground truth and the segmentation result, describing a measure of similarity. The definitions of *Accuracy, Precision, Recall*, and *F-score* are the same as those commonly used in machine learning. The explicit mathematical expressions of these metrics are shown in Table 1.

**Table 1:** Different evaluation indicators.

**Performance measure**	**Mathematical expression**
Jaccard index	$\frac{TP}{TP+FP+FN}$
Accuracy	$\frac{TP+TN}{TP+TN+FP+FN}$
Precision	$\frac{TP}{TP+FP}$
Recall	$\frac{TP}{TP+FN}$
Dice coefficient	$\frac{2\cdot TP}{2\cdot TP+FP+FN}$

#### 3.1.2. Segmentation Performance

Experiments on both datasets are conducted to compare with recent approaches.

On the ATUM-SEM dataset, we compared our method with U-Net (Ronneberger et al., 2015), Mask R-CNN and FFN. The same backbone network and feature pyramid network (FPN) are employed in Mask R-CNN. We note that we adopted a 2D version of FFN for this comparison for two main reasons: first, the ground truth is labeled on the 2D images in our dataset, which means the mitochondria cannot be discriminated from each other in 3D. However, the original FFN used a 3D reconstruction method that requires 3D segmentation for training. Second, our proposed network learns features only at the 2D level, but the original FFN trains a recurrent 3D convolutional network to extend objects; thus, it integrates 3D features. For a more consistent comparison, we trained a 2D CNN called FFN-2d, which has 9 convolution layers. The network architecture is generally the same as (Januszewski et al., 2018) except for the dimensions of the convolution operations. We set both the input and output sizes of FFN-2d to 33 × 33.

We present a quantitative comparison with state-of-the-art methods in Table 1 in terms of segmentation performance indicators, where the highest values are marked in bold for distinction. For a fair performance comparison, all the evaluation values were obtained directly from the networks, without any postprocessing. All the values for the thresholding operation were set to 127. As shown in Table 2, our proposed approach achieves a Jaccard index of 0.8021, a recall of 0.8442, and a dice coefficient of 0.8891. Its accuracy value (0.9876) is nearly equal to the highest value. Clearly, the proposed method achieves a better segmentation performance than do the previous methods. For qualitative comparison, two specific examples are illustrated in Figure 4. As indicated by the purple arrows, our proposed method produces more complete and accurate segmentation results than do the baseline approaches. From the detection viewpoint, our method obtains more accurate numbers of mitochondria.

**Table 2:** Segmentation performance comparison on the ATUM-SEM dataset.

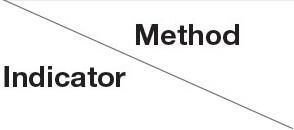	**U-Net**	**FFN-2d**	**Mask R-CNN**	**Proposed method**
Jaccard index	0.7972	0.6193	0.7799	**0.8021**
Accuracy	**0.9877**	0.9699	0.9863	0.9876
Precision	0.9403	0.7712	**0.9621**	0.9425
Recall	0.8397	0.7586	0.8051	**0.8442**
Dice coefficient	0.8845	0.7590	0.8750	**0.8891**

On the FIB-SEM dataset, we compared our method with Non-parametric Higher-order Random Fields (Márquez-Neila et al., 2014), Improved KernelBoost (Rigamonti et al., 2014), Kernelized SSVM/CRF (Lucchi et al., 2012a), and Lucchi2013 (Lucchi et al., 2013). In our method, the trained segmentation subnetwork outputs mitochondria probability maps for each slice. Then a simple thresholding operation is performed to obtain binary results for computing the jaccard index. Additionally, the network architecture and parameters are the same as those of ATUM-SEM dataset. For other methods, we obtain the reported values on the FIB-SEM dataset from corresponding literatures. The performance comparisons are shown in Table 3 in terms of Jaccard index. Our results yielded 0.864 Jaccard index, showing comparable performance with the other state-of-the-art methods.

**Table 3:** Segmentation performance comparison on the FIB-SEM dataset.

**Method**	**Jaccard index**
Kernelized SSVM/CRF	0.840
Lucchi2013	0.867
Non-parametric higher-order random fields	0.762
Improved KernelBoost	0.776
Ours proposed method	0.864

#### 3.1.3. Computational Costs

In this subsection, we present the time consumed by complete manual annotation and our proposed method to show the importance of having an automatic algorithm. Table 4 illustrates the time consumed for each step: the steps mainly consist of the ER segmentation and the segmentation of mitochondria.

**Table 4:** Computational costs comparisons between manual annotation and our proposed method.

	**Segmentation of ER**	**Segmentation of Mitochondria**
Manual annotation	4,044 h	5,055 h
Our proposed method	12.83 h (training) + 10.63 h (inference)	43.8 h (training) + 123 h (inference)

Due to the limited GPU memory of computers, we cut the original EM images into small patches used as network inputs. Therefore, all the computations are estimated based on the times required for these small patches. On average, it takes approximately 4 and 5 min to annotate all the ER and mitochondria in a 1,024 × 1,024 image, respectively, using the Trakem software. There are approximately 60,670 patches in the 28,628 μm^3^ EM stack. In our pipeline, the training time required 12.83 h for ER and 43.8 h for mitochondria. Subsequently, the inference step takes 0.6308 s for ER and 8.2081 s for the mitochondria in a 1,024 × 1,024 image. All the costs were measured on a Tesla K40 GPU. These times would be further accelerated with the application of more powerful computing resources. From the comparison, we can see that applying our method improves the segmentation costs by orders of magnitude.

### 3.2. 3D Reconstruction of Mitochondria

**Figure 7:**
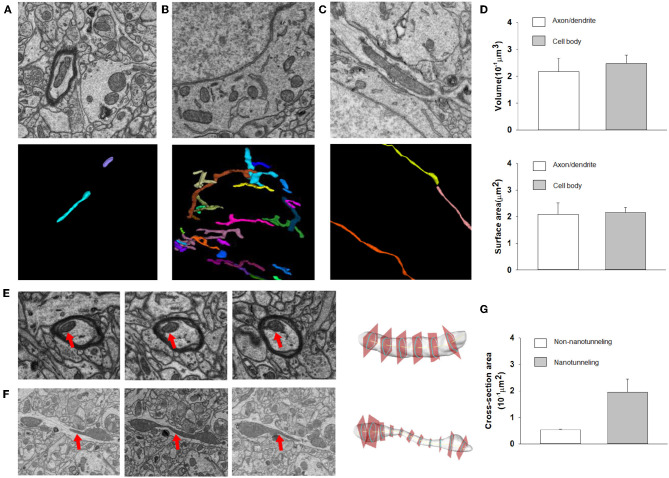
The 3D morphology of mitochondria for different neuron locations. **(A–C)** Raw EM images and 3D reconstructed mitochondria in axons, neuron somas and dendrites. **(D)** Top: The volumes of mitochondria in the cell body (*n* = 86) are greater than those in axons/dendrites (*n* = 81) (one-sided Mann–Whitney test, *p* < 0.0001). Bottom: The surface area of mitochondria in the cell body (*n* = 86) is greater than that in axons/dendrites (*n* = 81) (one-sided Mann–Whitney test, *p* = 0.0002). **(E,F)** Example of cross section analysis in non-nanotunneling and nanotunneling mitochondria, respectively. The red arrows indicate mitochondria. **(G)** The cross-sectional areas of non-nanotunneling and nanotunneling mitochondria.

After automatic segmentation and 3D reconstruction of the mitochondria of dendrites, axons and neurons in the mouse cortex, we found that mitochondrial morphology was varying in different cell positions (Figure 7). For example, in the axons and dendrites, the mitochondria were mainly long tubular structures (see Figure 7A). However, in the cell body, the mitochondria were typically adapted into reticular organizations (see Figures 7B,C). After quantification, the volume and surface area of the interconnected mitochondrial network in the cell body were approximately 0.217 and 2.093 μm^2^, respectively, which was larger than the mitochondrial networks in the axons and dendrites (see Figure 7D).

Previous studies indicated that communication between mitochondria involves adjacency (“kissing”) or a dynamic nanotubular tunnel between non-adjacent mitochondria (“nanotunneling”) (Sun et al., 2012; Huang et al., 2013). After automatic reconstruction of mitochondria in the mouse cortex, we found that non-nanotunneling communication was distributed mainly in axons/dendrites (Figures 7E,F). Next, we applied the cross-sectional area to detect and quantify the non-nanotunneling ones. As shown in Figure 7G, the average cross-sectional area is 0.053 and 0.194 μm^2^ in non-nanotunneling and nanotunneling mitochondria, respectively. We found that the cross-sectional area variance in nanotunneling mitochondria is 0.159 μm^2^, and 0.0058 μm^2^ in non-nanotunneling mitochondria. Our work revealed that automatic reconstruction of mitochondria provides a precise and unbiased method for detecting mitochondrial nanotunneling in neurons.

### 3.3. 3D Reconstruction of ER

**Figure 8:**
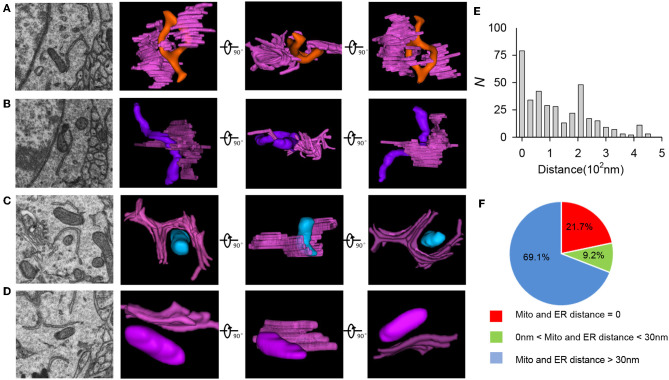
The 3D morphology of ER in neurons. **(A–D)** Left: the original EM image; Right: ER 3D morphology (pink) under different directions. **(E)** Mitochondria and ER distance distribution in neurons. **(F)** Percentage of different mitochondria and ER distances in neurons.

Mitochondria–ER contact sites are highly conserved structures in eukaryotic cells (Marchi et al., 2014). Several approaches have been described in the literature for quantifying the mitochondria–ER contact site distance from EM images (Filadi et al., 2015; Naon et al., 2016; Faustini et al., 2019; Garrido-Maraver et al., 2020). This distance has also been analyzed using 2D imaging of fluorescently labeled mitochondria and ER or 3D reconstruction of fluorescently labeled mitochondria and ER (Friedman et al., 2010; Rowland and Voeltz, 2012). Due to low resolution and artifacts in 2D analysis, previous researchers applied EM to study the relationship between mitochondria and ER, but they mainly used manual annotation to reconstruct mitochondria and ER, which is time consuming (Hirabayashi et al., 2017; Krols et al., 2018). Therefore, individual algorithms are needed to exactly detect the distance between mitochondria and ER in 3D space. In our work, we present a novel method for quantifying the distance between mitochondria and ER of neurons after 3D reconstruction using the ATUM-SEM technique. After automatic segmentation and reconstruction of ER in neurons, we found that the mitochondria and the ER have different structural features. As shown in Figure 8, apart from non-contact between these two organelles, some contacts between mitochondria and ER are more extensive. For example, we found that the ER tubules circumscribed almost completely around the mitochondrial membrane, either with (Figures 8A,B) or without direct contact (Figure 8C). The average distance between these two organelles was 133.499 nm. The percentage of direct contact between mitochondria and the ER is 21.7%, while the percentage of distances less than 30 nm but without direct contact between these two organelles is 9.2% (Figure 8F). Our method provides a precise and unbiased analysis of distances between mitochondria and ER with high resolution and no artifacts.

## 4. Discussion

The main function of mitochondria is to serve as a local energy source that supplies ATP (van der Bliek et al., 2017). Evidence suggests that mitochondrial morphology changes can lead to defects in intermitochondrial signal propagation, Ca^2+^ uptake, and neuronal protection against apoptotic stimuli (Karbowski and Youle, 2003; Bhatti et al., 2017). In this study, we developed an unbiased and rapid method for mitochondrial morphology detection. In the axons and dendrites, the mitochondria were mainly organized into long tubular structures. However, in the cytoplasm, the mitochondria were typically adapted into reticular networks. The extremely varied morphological features of mitochondria for a structural basis for maintaining energy homeostasis in neurons. A previous study reported that mitochondria are dynamically transported between axons and dendrites, which is crucial for synaptic and neuronal function (Wang and Schwarz, 2009; Zinsmaier et al., 2009). The elongated tubules of mitochondria in axons and dendrites might contribute to highly efficient mitochondrial transport. The high cross-sectional area SD of nanotunneling mitochondria in the axons and dendrites indicates a high ratio of fission and fusion processes, which benefits mitochondrial delivery among neuron bodies, axons, dendrites and synapses. Therefore, regional differences in mitochondrial morphological features are likely accompanied by organelle transport differences.

The contacts between mitochondria and ER are important for many physiological functions, such as apoptosis, calcium hemostasis, bioenergetics and protein synthesis (Marchi et al., 2014; Phillips and Voeltz, 2016). Many researchers documented that the function and structure of MAMs defects can result in multiple neurodegenerative diseases, such as Alzheimer's disease, Amyotrophic lateral sclerosis motor neuron disease, and Parkinson's disease (Manfredi and Kawamata, 2016; Liu and Zhu, 2017; Rodriguez-Arribas et al., 2017). Previous studies have documented the ER's role in mitochondrial fission and fusion. ER is involved in mitochondrial fusion and fission process (Friedman et al., 2011; Lee et al., 2016; Abrisch et al., 2020). Our work provided a precise and unbiased method to detect the mitochondria and ER relationship in 3D space with high resolution. The 3D reconstruction of these two organelles can provide structural evidence. Due to low resolution of fluorescent microscopy and artifacts in 2D analysis, large-scale EM provides an excellent approach to study and quantify the contacts between these two organelles. Using high-resolution 3D reconstruction, ER tubules were shown to wrap around the mitochondria in neurons. Our data confirms that ER tubule may be involved in mitochondria division machinery recruitment in the early stage. Meanwhile, our work shows ER may play an unexpected role during the mitochondrial biogenesis process.

This paper proposes an automated pipeline that effectively obtains the 3D morphology of mitochondria, ER and mitochondria-ER contact sites in large-scale ATUM data. To enable the 3D segmentation of mitochondria, we first obtain the instance segmentations on each slice; then, we connect the 2D mitochondria to obtain the 3D reconstruction results based on contextual information. Instance segmentation is achieved by exploiting a recurrent network that outperforms most state-of-the-art methods. Compared with a semantic segmentation method, utilizing a region-based strategy can produce instance segmentation directly while preventing interruptions from noise and artifacts. We added a recursive segmentation subnetwork to refine the segmentation results, which reduces the effect of inaccurate detection results. In the case of the ER and nuclear membrane, 3D connections can be obtained simply in a single cell body. Therefore, we utilize a fully CNN to learn the ER and nuclear membrane as a single membrane system that are subsequently distinguished using a relabeling method.

## Data Availability Statement

The datasets for this article are not publicly available because the datasets are being used for other studies. Requests to acess the datasets should be directed to yangyang2@shanghaitech.edu.cn.

## Ethics Statement

The animal study was reviewed and approved by Animal Committee of the Institute of Neuroscience Chinese Academy of Sciences.

## Author Contributions

JL and LL designed the experiments and wrote the paper. YY conducted the sample preparation. LL provided the scanning electron microscope images. BH conducted the annotation and visualization. XC registered the EM images. HH and QX conceived the ideas for all the methods and provided suggestions based on the experimental results.

## Conflict of Interest

The authors declare that the research was conducted in the absence of any commercial or financial relationships that could be construed as a potential conflict of interest.
